# Multiplex CRISPR-Cas9 knockout of *EIL3, EIL4*, and *EIN2L* advances soybean flowering time and pod set

**DOI:** 10.1186/s12870-023-04543-x

**Published:** 2023-10-27

**Authors:** Yunqing Cheng, Yujie Li, Jing Yang, Hongli He, Xingzheng Zhang, Jianfeng Liu, Xiangdong Yang

**Affiliations:** 1 Jilin Provincial Key Laboratory of Plant Resource Science and Green Production, Jilin Normal University, Siping, Jilin Province, 136000 China; 2 Jilin Provincial Key Laboratory of Agricultural Biotechnology, Jilin Academy of Agricultural Sciences, Changchun, 130024 China

**Keywords:** Soybean, Ethylene, *EIL*, *EIN2*, CRISPR-cas9

## Abstract

**Background:**

Ethylene inhibitor treatment of soybean promotes flower bud differentiation and early flowering, suggested that there is a close relationship between ethylene signaling and soybean growth and development. The short-lived ETHYLENE INSENSITIVE2 (*EIN2*) and ETHYLENE INSENSITIVE3 (*EIN3*) proteins play central roles in plant development. The objective of this study was carried out gene editing of *EIL* family members in soybeans and to examine the effects on soybean yield and other markers of growth.

**Methods and results:**

By editing key-node genes in the ethylene signaling pathway using a multi-sgRNA-in-one strategy, we obtained a series of gene edited lines with variable edit combinations among 15 target genes. *EIL3*, *EIL4*, and *EIN2L* were editable genes favored by the T0 soybean lines. Pot experiments also show that the early flowering stage R1 of the *EIL3, EIL4*, and *EIN2L* triple mutant was 7.05 d earlier than that of the wild-type control. The yield of the triple mutant was also increased, being 1.65-fold higher than that of the control. Comparative RNA-seq revealed that sucrose synthase, AUX28, MADS3, type-III polyketide synthase A/B, ABC transporter G family member 26, tetraketide alpha-pyrone reductase, and fatty acyl-CoA reductase 2 may be involved in regulating early flowering and high-yield phenotypes in triple mutant soybean plants.

**Conclusion:**

Our results provide a scientific basis for genetic modification to promote the development of earlier-flowering and higher-yielding soybean cultivars.

**Supplementary Information:**

The online version contains supplementary material available at 10.1186/s12870-023-04543-x.

## Background

Ethylene plays an important regulatory role in plant growth and development, particularly in response to biotic and abiotic stress. A linear model of ethylene signal transduction has been established by screening the ethylene "triple response" mutants [1]. In this model, *CTR1* is a negative regulator of the ethylene signal transduction pathway and phosphorylates *EIN2* after binding with the receptor. The phosphorylation-regulated proteolytic processing of *EIN2* triggers its trafficking from the endoplasmic reticulum (ER) to the nucleus. Ethylene triggers dephosphorylation at several sites and proteolytic cleavage at one of these sites, resulting in nuclear translocation of a carboxyl-terminal *EIN2* fragment (EIN2-C'). The EIN2-C' peptide combines with *EIN3* to jointly regulate the downstream target genes [2], which further activates downstream ethylene-related transcription factors, such as *ERF1*, and amplifies the ethylene signal [3]. In the ethylene signal transduction pathway, the EIN2-C' peptide combines with transcription factors ETHYLENE INSENSTIVE 3 (*EIN3*) and ETHYLENE INSENSITIVE 3-LIKE 1 (*EIL1*) to positively regulate the ethylene signaling pathway [2].

*EIN2* and *EIN3/EIL* have been identified in many plants. For instance, *EIN2* and *EIN3/EIL* affect seedling growth and responses to salt and other stressors in Arabidopsis [4–7]. In Petunia, reduced *PhEIN2* expression delays flower senescence and fruit ripening, and reduces adventitious root and seedling root-hair formation [8]. In rice, homologous genes of Arabidopsis ethylene signaling components have been identified, including *ETR*, *RTE1-like*, *EIN2-like*, and *EIN3-like* genes, which control grain size and weight, root growth, salt tolerance, and wound signaling [9–12]. In soybean, comparative transcriptome analysis has shown that exogenous ethephon treatment might activate *EIN3* and *ERF1/2* in the ethylene signal transduction pathway, amplify ethylene signaling, inhibit soybean flowering, and promote flowering reversal. Ethylene inhibitor treatment of soybean inhibits the transcriptional activity of *ERF1/2*, promotes flower bud differentiation, and leads to early flowering [13–15]. Although genome-wide analyses of the *EIN2* and *EIN3/-EIL* gene family has been carried out for several plant species, no comprehensive analysis of this family has been reported for soybean. Since the soybean is a globally important agricultural crop, the *EIL* gene family should be thoroughly investigated in its biology.

To characterize the function of *EIN2* and *EIN3/EIL* gene family members in plant development, the mutation phenotype and overlapping, opposite or different functions of *EIN2* and *EIN3/EIL* gene family members should be analyzed. The ethylene-response phenotype in Arabidopsis *ein3* mutant was lost due to the reduced expression of *ERF1* and chitinase genes but the mutant phenotype could be rescued by the overexpression of *EIL1* or *EIL2*, which indicates that *EILs* play an important regulatory role in soybean ethylene signaling pathway [16, 17]. *LeEILs* members are functionally redundant and played vital role in regulating ethylene reaction during the whole life cycle of tomato. However, genetic mutant screens of *EIL* family members in soybean are limited due to its paleopolypoid genome. Another major obstacle in the gene editing application of CRISPR-Cas9 for soybean is its low transformation efficiency. The key components in the ethylene pathway contain several gene family members, and, therefore, knocking out subjectively and randomly only a single gene would lead to weaker phenotypic differences. Fortunately, multiplex mutations can now be generated by either cloning multiple sgRNAs or targeting multiple homologous regions by a single sgRNA in the CRISPR-Cas9 system in both dicot and monocot plants [18].

In our study, one pYLCRISPR-Cas9 plasmid with 15 sgRNA cassettes for editing 15 *EIN2* and *EIN3*/*EIL* genes was constructed for soybeans guided by natural variation. The screening of mutants followed by cultivation experiments provided useful information for the selection of *EIN2* and *EIN3*/*EIL* genes that are agronomically important.

## Results

### Phylogenetic analysis of EIN2 and EIL

**Fig. 1:**
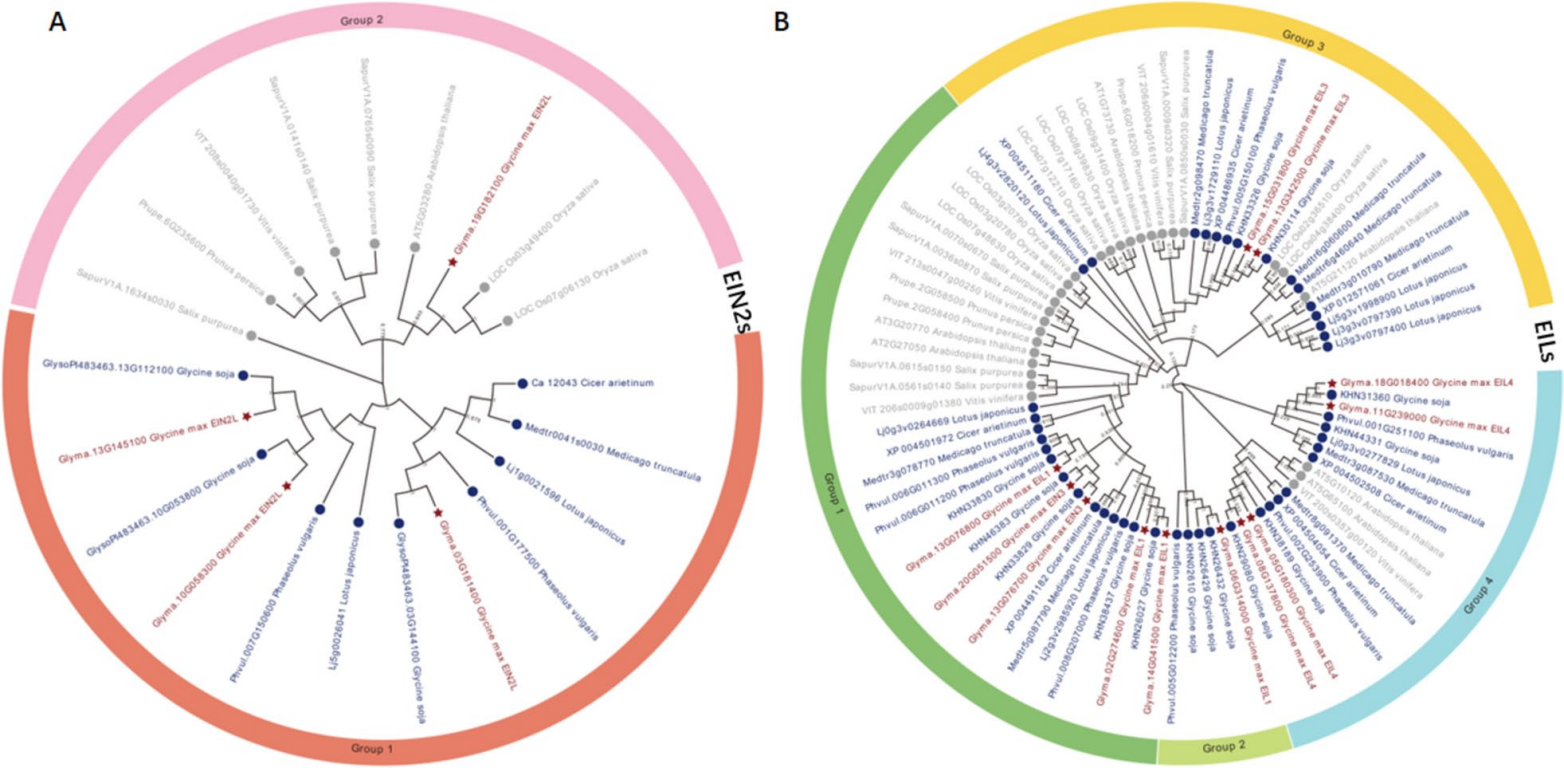
Phylogenetic tree of *EIN2* and *EILs* proteins in plants. **A** Phylogenetic tree of *EIN2s* in soybean cultivar Williams 82. **B** Phylogenetic tree of *EILs* in soybean cultivar Williams 82. Red font indicates 12 *EILs* and 4 *EIN2* proteins in soybean cultivar Williams 82

A total of 260 *EIN2s* and 1425 *EILs* were found in Ensembl Plants (http://plants.ensembl.org/) respectively. There were three *EIN2s* and eight *EILs* in genome of *Arabidopsis thaliana*. We identified 12 *EIL* and four *EIN2* genes in the soybean W82 genome. Compared to Arabidopsis, soybean has more *EIN2s* and *EILs* members, which is consistent with the more complex paleopolypoid genome of soybean. To explore the phylogenetic relationships of the *EIN2* and *EIL* families, a neighbor-joining (NJ) tree was constructed by aligning the sequences with 89 other sequences from *A. thaliana*, *Cicer arietinum*, *Glycine soja*, *Lotus japonicus*, *Medicago truncatula*, *Oryza sativa*, *Phaseolus vulgaris*, *Prunus persica*, *Salix purpurea*, and *Vitis vinifera* (Fig. 1A and B). The results of systematic evolution analysis showed that the 21 *EIN2s* and 84 *EILs* from different plants were divided into two and four evolutionary branches, respectively (Fig. 1A and B). Four *EIN2* genes belonged to two evolutionary branches, indicating that they may come from the two ancestral genes (Fig. 1A). All *EILs* were divided into four evolutionary branches, each with five, one, two, and four members, indicating that these 12 *EIL* genes may come from four ancestral genes (Fig. 1B). The results also showed that most of 12 *EIL* and four *EIN2* gene members from *G. max* had a relatively close genetic relationship with *G. soja*, *L. japonicus*, and *P. vulgaris*, while had a relatively distant genetic relationship with *A. thaliana* and *O. sativa.*

### CRISPR-Cas9 system for targeted mutagenesis of EIN2 and EIL genes in soybean

**Fig. 2:**
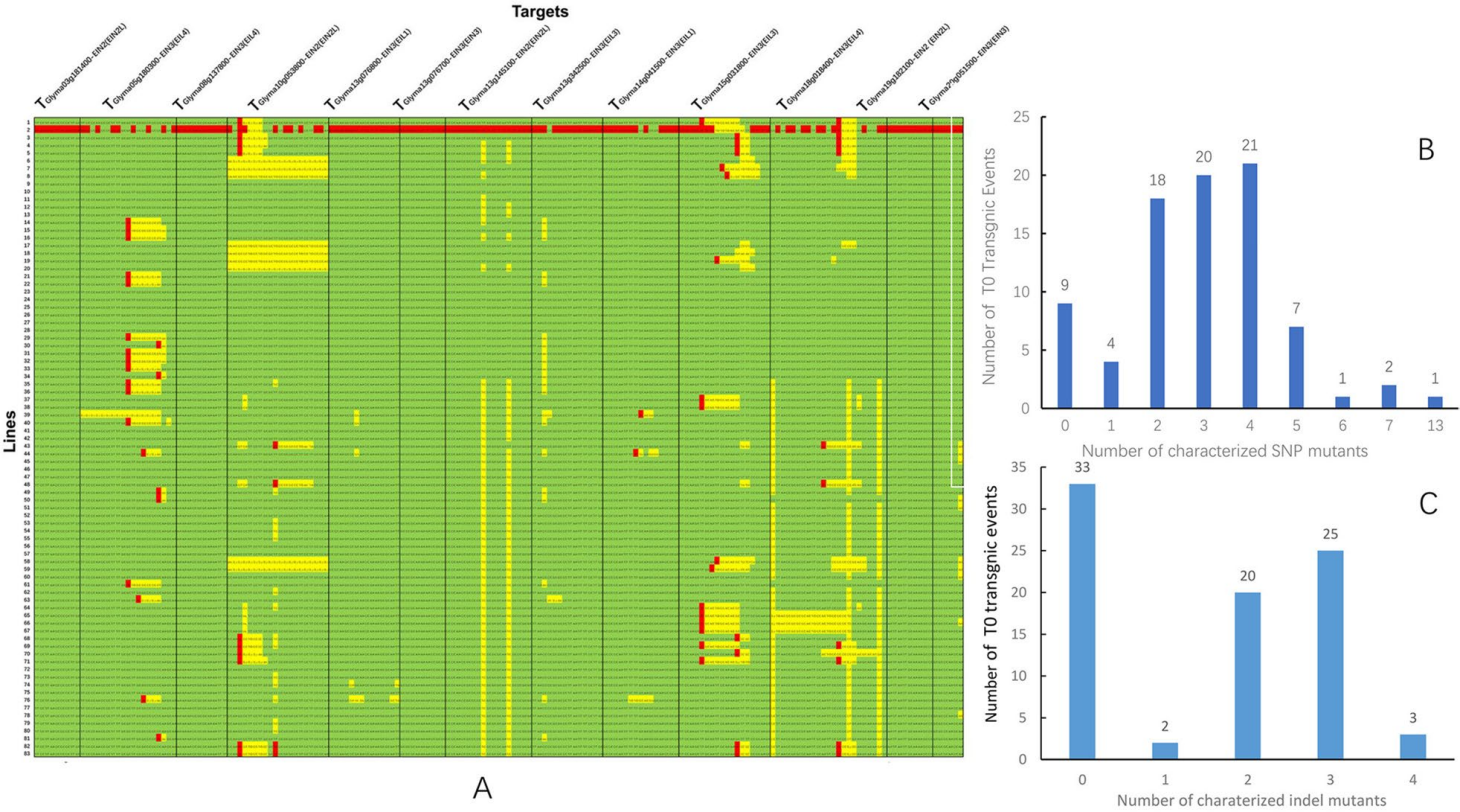
Comparative assays of single-nucleotide polymorphism/insertion or deletion (SNP/InDel) in the T0 soybean using the CRISPR/Cas9 gene editing technology. **A** Single-nucleotide polymorphisms and haplotype analysis in *EILs* and *EIN2* genes in different T0 lines. **B** Number of characterized SNP mutant in T0 gene editing events. **C** Number of the characterized indel mutant in T0 gene editing events

The CRISPR-Cas9 vector system for multiplex targeting of genomic sites was used to clone the target sequences of 15 genes into one vector and carry out genetic transformation. The whole genomic DNA of transformed plants was analyzed by PCR and sequencing. Combining with PAT/Bar test strip detection, 83 T-DNA positive plants were obtained in the T0 generation, and further sequencing of the gene targets in the transformed plants showed that 13 genes targets were successfully edited in the 74 T0 generation lines. All the 13 target genes were successfully knocked out in one plant, which caused arrested growth and development (Fig. 2A and B). Among the 83 glufosinate-resistant T0 plants, two, three, and four SNP mutations had the highest frequencies in 18, 20, and 21 plants, accounting for 71.08% of the total glufosinate-resistant T0 plants (Fig. 2B). Among all mutated plants, the number of plants without indel mutations was the highest, accounting for 39.76% of the total; among plants with indel mutations, the number of plants with two or three indel mutations was the highest, accounting for 54.22% of the total (Fig. 2C). Sequencing the 13 target genes in 74 positive T0 plants confirmed simultaneous editing of *EIL3* (*Glyma.15G031800*), *EIL4* (*Glyma.18G018400*), and *EIN2L* (*Glyma.10G058300*) in 23 lines. This suggests that these three genes are preferred editable genes in soybean, and their simultaneous editing may be conducive to cell reproduction.

### EIL3, EIL4 and EIN2L triple mutants

**Fig. 3:**
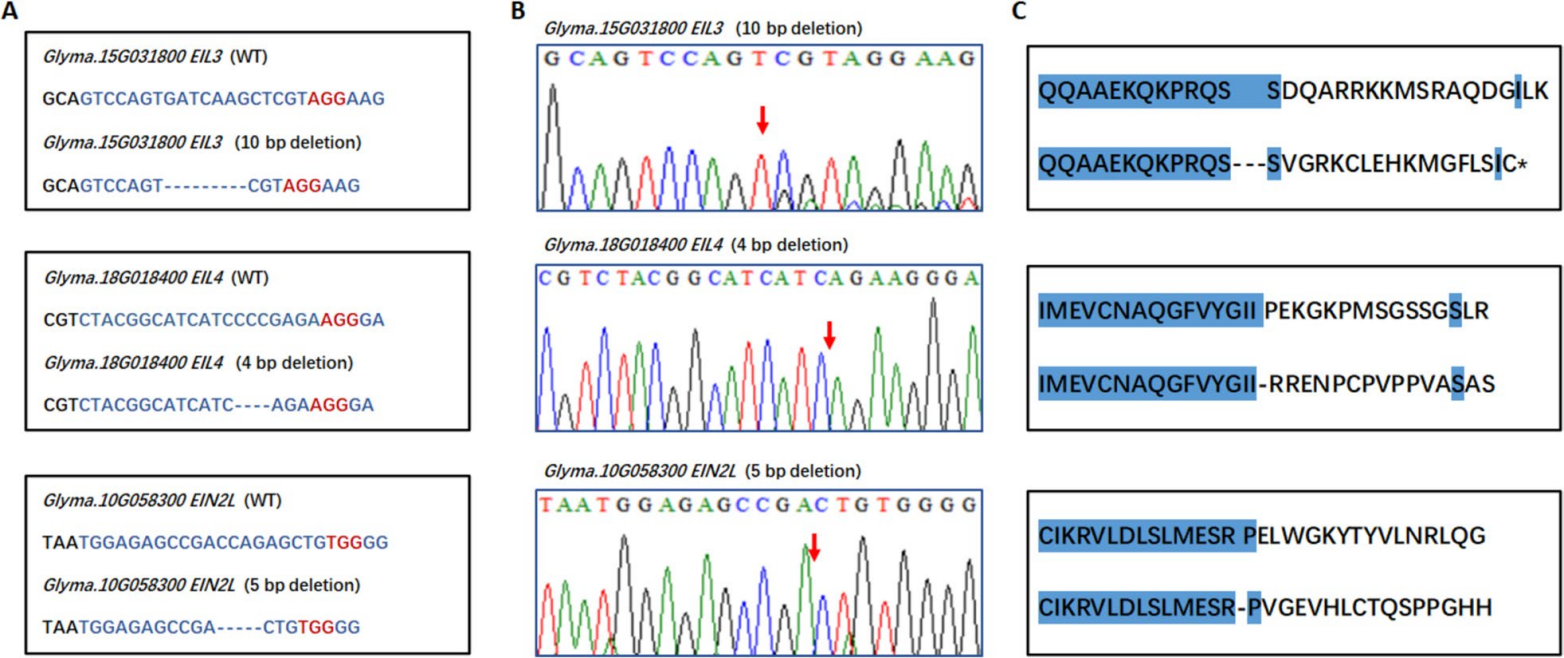
Soybean triple mutation of *EIL3, EIL4* and *EIN2L* generated by the CRISPR/Cas9 system in the T3 generation. **A** Sequences of WT and mutant plants at target sites *EIL3*-sg-T1, *EIL4*-sg-T2, and *EIN2L*-sg-T3. Dashes indicate deleted nucleotides. Proto-spacer adjacent motif (PAM) has been highlighted with red letters. **B** The sequence peaks of the wild-type (WT) and mutants at target sites *EIL3*-sg-T1, *EIL4*-sg-T2, and *EIN2L*-sg-T3. Red arrows indicate mutation locations. **C** Amino acid sequences of the triple mutation of *EIL3, EIL4* and *EIN2L* and WT

In *EIN2L*, *EIL3*, and *EIL4* triple mutant lines (abbreviated as experiment number Z4) of T2 generation, we identified Cas9-free plants carrying targeted mutations in which the T-DNA was lost while the *EIN2L*, *EIL3*, and *EIL4* genes were simultaneously edited. Sequencing of the *EIL3*, *EIL4*, and *EIN2L* T3-mutant plants revealed a 10-bp deletion at T1 + sgRNA-targeted *EIL3*, a 4-bp deletion at T2 + sgRNA-targeted *EIL4,* and a 5-bp deletion at T3 + sgRNA-targeted *EIN2L* (Fig. 3A and B), resulting in frameshift mutations in the *EIL3*, *EIL4*, and *EIN2L* proteins (Fig. 3C)*.* The editing sites of *EIL3* and *EIL4* were at the 5' end of the gene, indicating that most of the amino acids in their proteins were mutated from the N' end. The editing sites of *EIN2L* were biased towards the 3' end, indicating that the EIN2-C' peptide of its protein was altered.

### Phenotypic changes in triple mutant plants

**Fig. 4:**
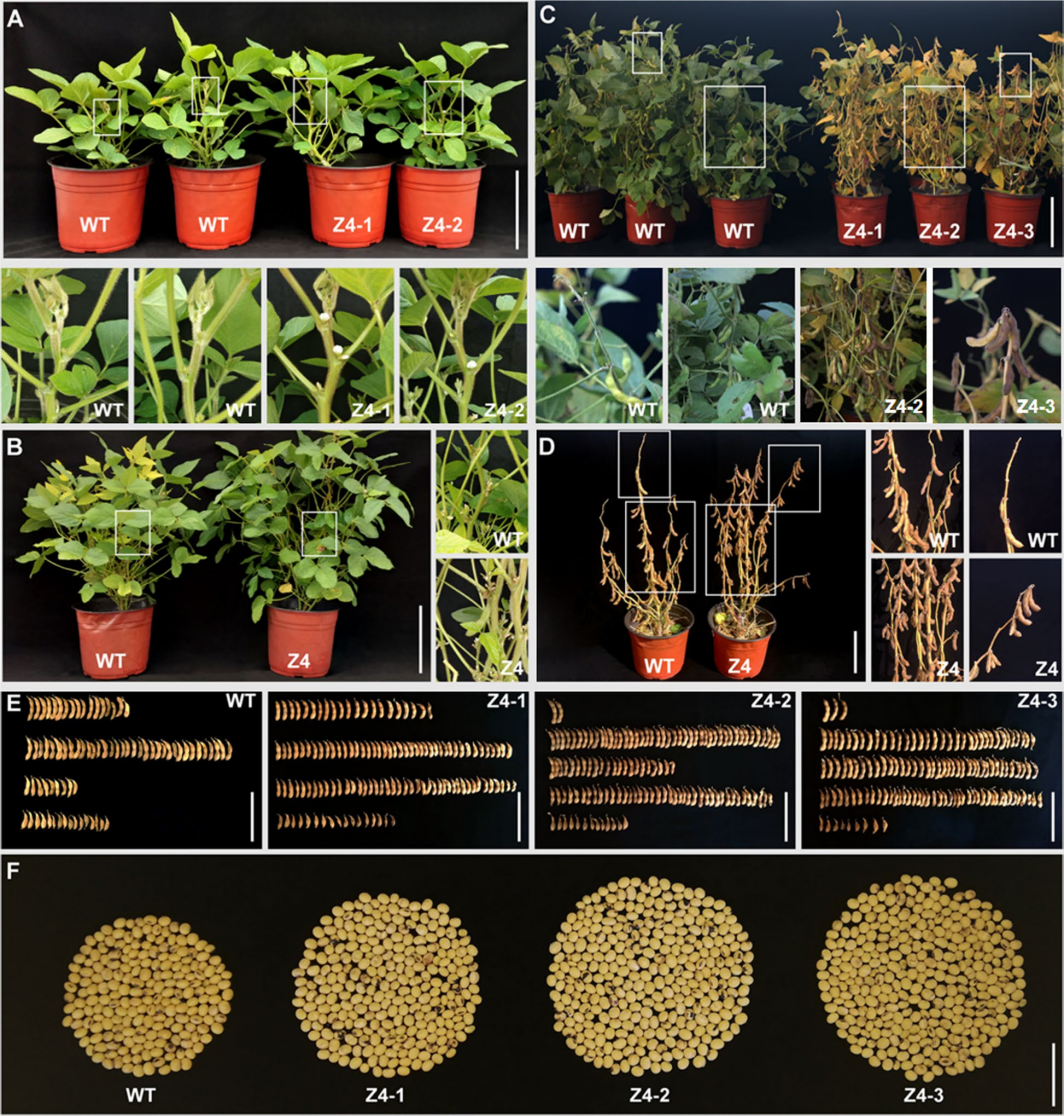
Phenotypes of EIL3, EIL4 and EIN2L triple mutant Z4 and WT. **A** Triple mutation leads to early flowering; **B** Triple mutation leads to early podding; **C** Triple mutation leads to premature pods; **D** Triple mutation plants and control at harvest; **E** Pods of triple mutation plants and control; **F** Seeds of triple mutation plants and control. WT, wild type; Z4, triple mutation plant. Z4-1, -2 and -3 are three replicate plants from the Z4 triple mutant line

**Fig. 5:**
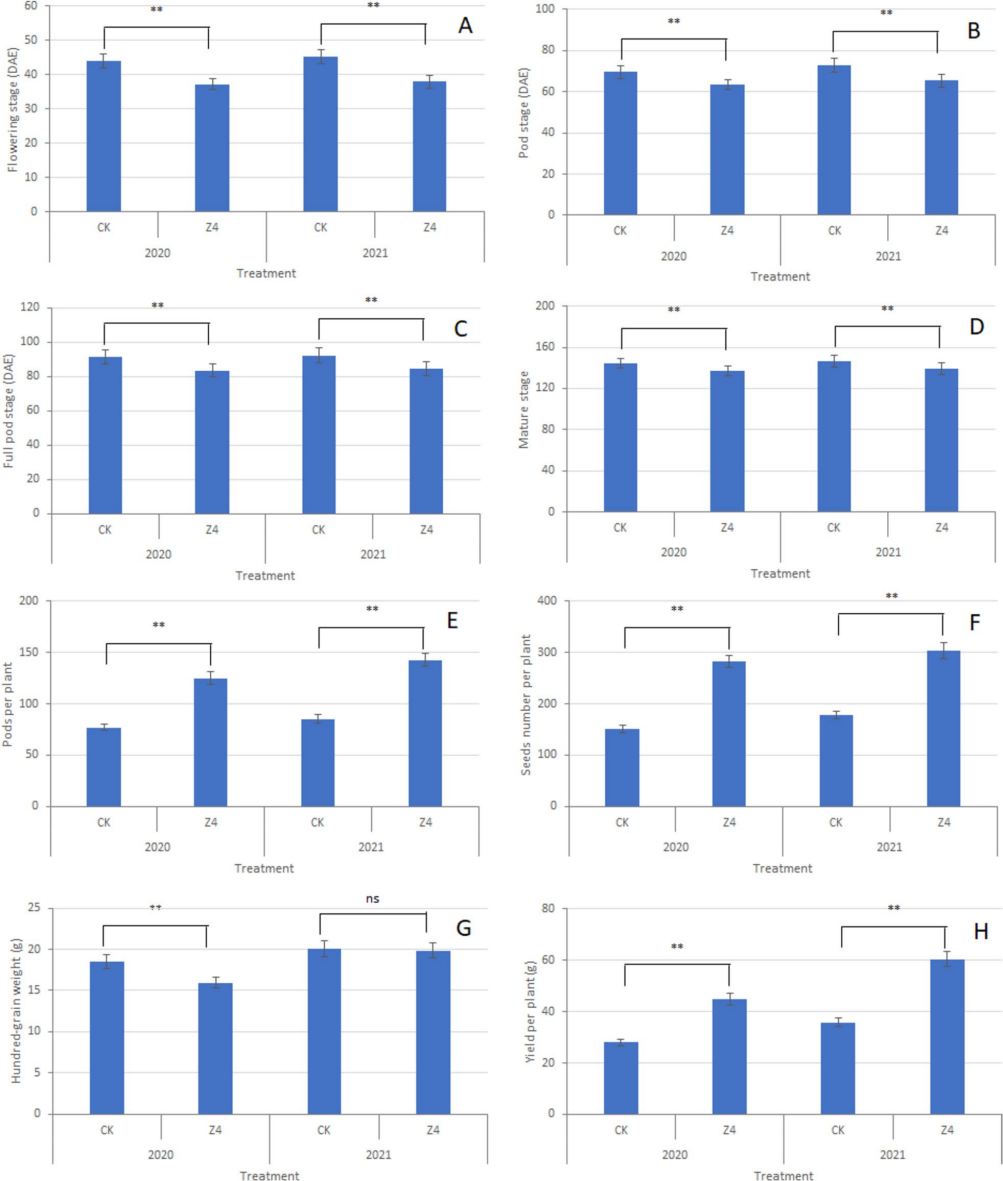
Growth period and pod setting habit changes of triple mutant. **A** Flower stage changes; **B** Pod stage changes; **C** Full pod stage changes; **D** Mature stage; **E** Changes of pods per plant; **F** Changes of seeds number per plant; **G** changes of 100 grain weight; **H** changes of yield per plant. DAE, days after emergence. CK, WT of soybean W82; Z4, triple mutation plant. All physiological indexes were measured for at least 10 individual plants. Means were compared using LSD (least significant difference) t-test at the 5% level of significance. ** represents significant difference at *P* ≤ 0.05 as indicated by LSD test

Compared to that of the wild-type control, the early flowering stage R1 of Z4 was 7 d earlier (Figs. 4A and 5A), the early podding stage R3 of the triple mutant was 6 d earlier (Figs. 4B and 5B), the full pod stage R4 of the triple mutant was 8 d earlier (Fig. 5C), and the maturation stage R8 of the triple mutant was 7 d earlier (Figs. 4C and 5D). At the maturity stage R8, all leaves were removed and the differences in pod-setting characteristics between the two treatments were compared. Compared to the Z4 plants, fewer pods formed on the top branches of the control plants (Fig. 4D). Furthermore, the average number of pods per plant was 133.65 for the Z4 plants compared to 81.18 for the wild-type controls (Figs. 4E and 5E). At harvest, an average of 293.29 beans were harvested per Z4 plant compared to 164.69 for each control plant, and the number of seeds per plant was also 1.78-fold higher in the Z4 plants (Fig. 5F). The average 100-grain weight of the Z4 plants was 17.89 g compared to 19.28 g for the wild type (Fig. 5G), and the yield of Z4 plant was 52.67 g compared to 31.85 g for the wild-type plant (Figs. 4F and 5H). Overall, the *EIL3*, *EIL4,* and *EIN2L* triple mutant had a significantly accelerated growth rate, improved pod-setting at the top of the plants, and significantly increased yield by 65% compared with the wild-type control plants.

### Identification of differentially expressed transcripts

**Fig. 6:**
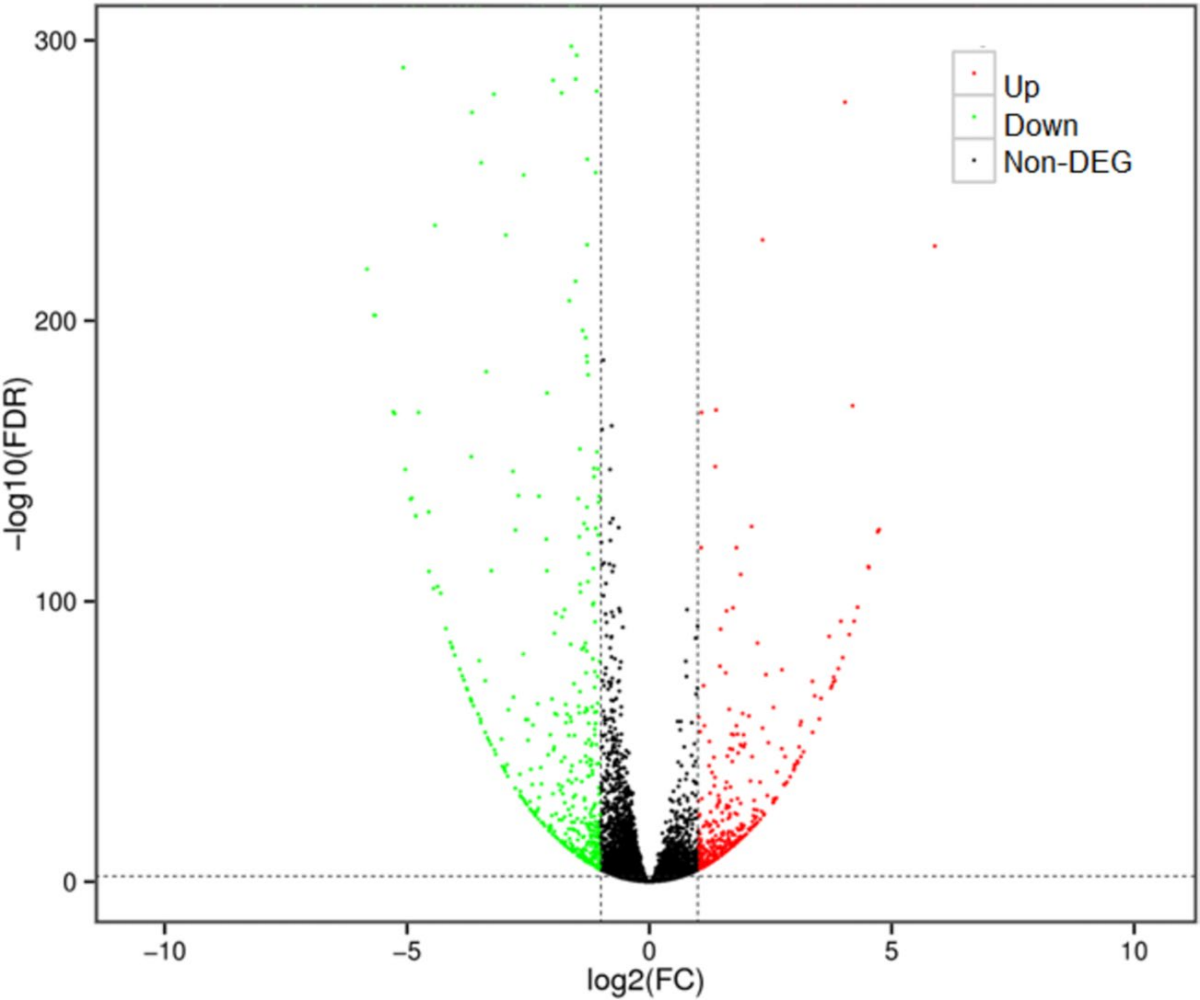
Volcano map of differentially expressed genes between control and triple mutation plants. Note: Each point in the volcano map represents a gene, and the abscissa represents the logarithm of expression fold change of a gene in the two samples. The ordinate represents the negative logarithm of the statistical significance of the change in gene expression. The greater the absolute value of abscissa, the greater the difference of expression multiple between the two samples. The larger the ordinate value, the more significant the differential expression, and the more reliable the differentially expressed genes screened. A fold change (FC) ≥ 2 and false discovery rate (FDR) < 0.01 was used to identify DEGs. Up or down-regulated genes refer to those in the Z4 triple mutant relative to WT control plants. Green dots represent down regulated differentially expressed genes, red dots represent up regulated differentially expressed genes, and black dots represent non differentially expressed gene (non-DEG)

After sequencing data quality control, six digital gene expression (DGE) libraries were generated composed of 169,592,355 clean reads covering a length of 50,498,587,958 clean bases (Table S1). Each clean read was 298-bp long. The obtained clean reads were mapped to the W82 genome, and the mapping ratio ranged from 95.63% to 96.16% (Table S2). All raw transcriptome data were deposited in the sequence read archive (accession no. PRJNA774476). The mapped reads were assembled and aligned to the reference genome to obtain annotations. Differentially expressed genes (DEGs) were identified using DESeq2 according to a fold change (FC) ≥ 2 and false discovery rate (FDR) < 0.01. Compared to the WT, 412 upregulated and 461 downregulated unigenes were identified in the triple mutant (Fig. 6; Table S3 and S4).

### KEGG enrichment analysis of differentially expressed genes

**Fig. 7:**
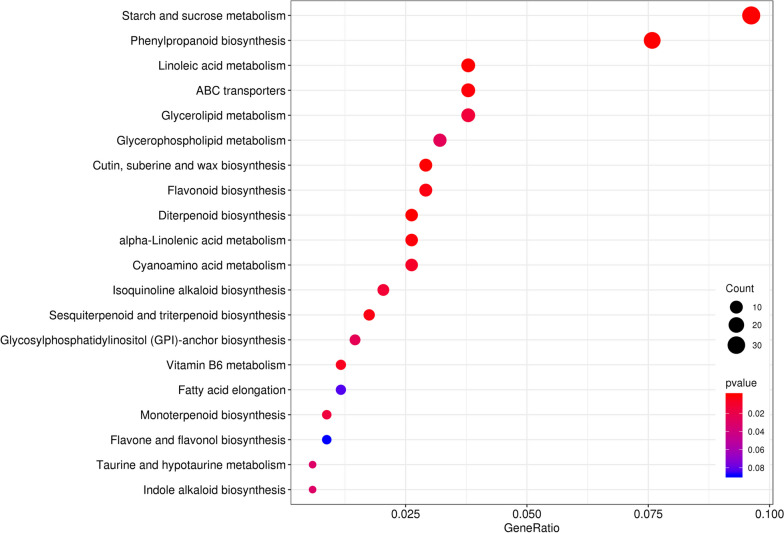
Significantly enriched pathways in control and triple mutation plants paired comparisons. Note: *P*-value ≤ 0.05 is set as the significant threshold for significantly enriched GO terms

A total of nine KEGG pathways were significantly enriched, with Q-values smaller than 0.05 (Fig. 7; Table S5). These DEGs were associated with linoleic acid metabolism (ko00591); starch and sucrose metabolism (ko00500); cutin, suberine, and wax biosynthesis (ko00073); phenylpropanoid biosynthesis (ko00940); diterpenoid biosynthesis (ko00904); ABC transporters (ko02010); alpha-linolenic acid metabolism (ko00592); flavonoid biosynthesis (ko00941); and sesquiterpenoid and triterpenoid biosynthesis (ko00909). Most of the significantly enriched KEGG pathways were related to metabolism and biosynthesis, suggesting that the expression of these genes in the Z4 flower organ may be responsible for its earlier flowering and high-yielding phenotype.

### DEGs in plant hormone signal transduction pathways

*EIL3*, *EIL4*, and *EIN2L* are important gene members in ethylene signal transduction and their knockout may have important impacts on plant hormone signaling due to crosstalk effects. The editing of these three genes had no significant effect on ethylene signaling pathway (Figure S1; Table 1). In the triple mutant, nine genes involved in auxin signaling were up-regulated 1 to 1.8-fold (Figure S1; Table 1). This included two *AUX1* genes (*Glyma.03G063900* and *Glyma.13G319700*), five *Aux/IAA* genes (*Glyma.02G142400*, *Glyma.07G018100*, *Glyma.07G034200*, *Glyma.19G161100*, and *Glyma.20G210400*) and two *SAUR* genes (*Glyma.08G155700* and *Glyma.12G034100*). In the cytokinin signal transduction pathway, a DEG (*Glyma.03G241700*) encoding *AHP* was upregulated by 1.03-fold, and two DEGs (*Glyma.05G158900* and *Glyma.08G116700*) encoding *B-ARR* were downregulated by 1.12-fold and 1.17-fold, respectively (Figure S1; Table 1). In the jasmonic acid signal transduction pathway, four *JAZ* genes (*Glyma.01G204400*, *Glyma.11G038600*, *Glyma.13G112000*, and *Glyma.13G116100*) and *MYC2* (*Glyma.15G166800*) was also downregulated by 1.14 to 2.50-fold (Figure S1; Table 1). In the salicylic acid signal transduction pathway, a *TGA6* (*Glyma.09G184300*) was downregulated by 4.93-fold (Figure S1; Table 1). In the brassinosteroid signal transduction pathway, five *BRI1* genes (*Glyma.06G184400*, *Glyma.18G254000*, *Glyma.18G254500*, *Glyma.18G254600*, and *Glyma.19G081200*) were upregulated by 1.22 to 2.08-fold, while *SBP13* and *SBP1* (*Glyma.11G083900* and *Glyma.13G177400*) were downregulated by 1.25 and 1.24-fold (Figure S1; Table 1). Based on these results, gene editing of *EIL3*, *EIL4*, and *EIN2L* may influence the signal transduction pathways of auxin, cytokinin, jasmonic acid, salicylic acid, and brassinosteroids.

**Table 1:** DEGs in plant hormone signal transduction pathways

Gene ID	FDR	Log_2_FC	Annotation	Signaling pathway
*Glyma.03G063900*	2.14E-13	1.04	*AUX1*	auxin
*Glyma.13G319700*	2.28E-11	1.07	*AUX1*	auxin
*Glyma.02G142400*	3.69E-07	1.07	*Aux/IAA*	auxin
*Glyma.07G018100*	1.65E-05	1.01	*Aux/IAA*	auxin
*Glyma.07G034200*	1.99E-13	1.00	*Aux/IAA*	auxin
*Glyma.19G161100*	6.50E-120	1.80	*Aux/IAA*	auxin
*Glyma.20G210400*	3.57E-45	1.33	*Aux/IAA*	auxin
*Glyma.08G155700*	1.07E-05	1.03	*SAUR*	auxin
*Glyma.12G034100*	3.66E-05	1.03	*SAUR*	auxin
*Glyma.03G241700*	1.28E-05	1.03	*AHP*	cytokinin
*Glyma.05G158900*	1.94E-17	-1.17	*B-ARR*	cytokinin
*Glyma.08G116700*	4.48E-17	-1.12	*B-ARR*	cytokinin
*Glyma.01G204400*	3.29E-51	-2.50	*JAZ*	jasmonic acid
*Glyma.11G038600*	9.66E-48	-1.97	*JAZ*	jasmonic acid
*Glyma.13G112000*	4.38E-24	-1.14	*JAZ*	jasmonic acid
*Glyma.13G116100*	4.23E-104	-1.42	*JAZ*	jasmonic acid
*Glyma.15G166800*	3.06E-08	-1.20	*MYC2*	jasmonic acid
*Glyma.09G184300*	5.03E-137	-4.93	*TGA6*	salicylic acid
*Glyma.06G184400*	3.94E-19	2.08	*BRI1*	brassinosteroid
*Glyma.18G254000*	3.51E-09	1.42	*BRI1*	brassinosteroid
*Glyma.18G254500*	1.28E-10	1.53	*BRI1*	brassinosteroid
*Glyma.18G254600*	5.28E-08	1.27	*BRI1*	brassinosteroid
*Glyma.19G081200*	2.54E-21	1.22	*BRI1*	brassinosteroid
*Glyma.11G083900*	1.14E-08	-1.25	*SBP13*	brassinosteroid
*Glyma.13G177400*	5.57E-20	-1.24	*SBP1*	brassinosteroid

The character "*glyma.Wm82.gnm2.*" are omitted before gene IDs. FDR, false discovery rate; FC, fold change: FPKM of triple mutants divided by FPKM of control; *AHP*, *histidine-containing phosphotransfer protein*; *Aux/IAA*, *auxin-responsive protein IAA*; *AUX1*, *auxin influx carrier*; *B-ARR*, *two-component response regulator ARR-B family*; *BRI1*, *protein brassinosteroid insensitive 1*; *JAZ*, *jasmonate ZIM domain-containing protein*; *MYC2*, *transcription factor MYC2*; *SAUR*, *SAUR family protein*; *SBP, squamosa promoter binding like protein*; *TGA, transcription factor TGA*. The complete annotations for each DEG are listed in Table S4

### Validation of DEGs and non-DEGs by qRT-PCR

**Fig. 8:**
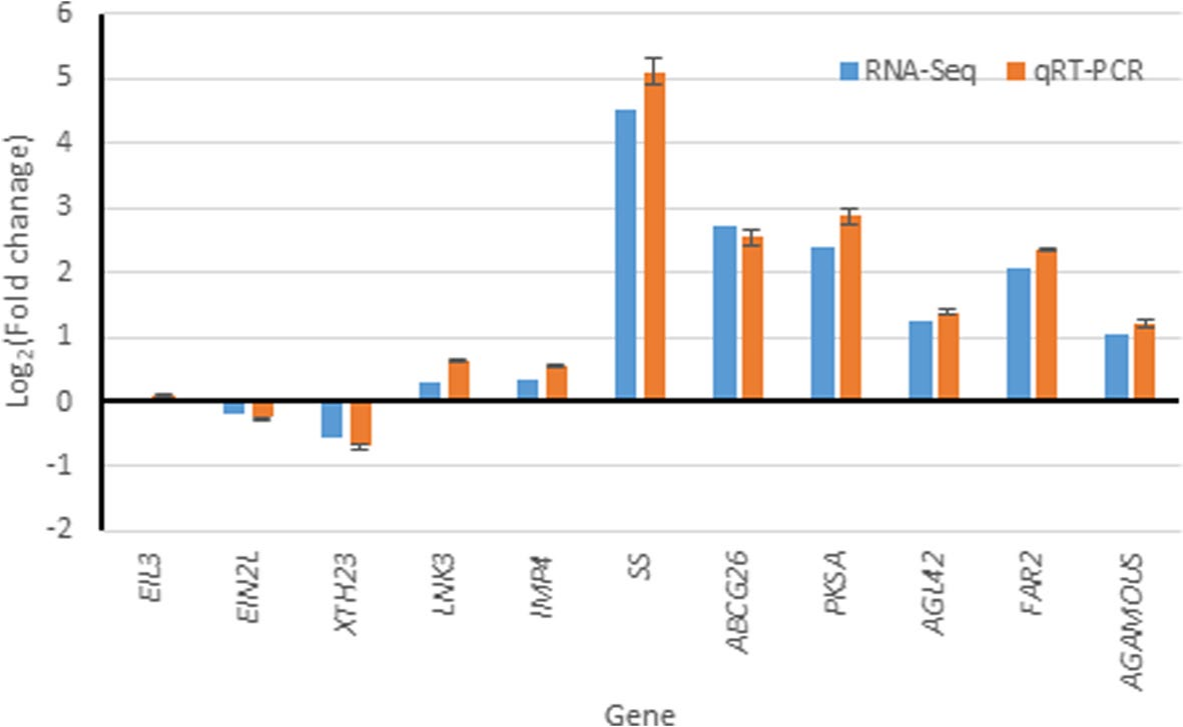
Validation of non-DEGs and DEGs by qRT-PCR analysis. Bars represent mean ± standard deviation (*n* = 3). The significance level was set as *P* < 0.05. Note: *EIL3, Ethylene insensitive 3*; *EIN2L, Ethylene insensitive 2 like*; *XTH23, xyloglucan endotransglucosylase/hydrolase protein 23*; *LNK3, Protein LNK3; IMP4, U3 small nucleolar ribonucleoprotein protein IMP4; SS, Sucrose synthase*; *ABCG26, ABC transporter G family member 26*; *PKSA, Type III polyketide synthase A*; *AGL42, MADS-box protein AGL42*; *FAR2, Fatty acyl-CoA reductase 2*; *AGAMOUS, Floral homeotic protein AGAMOUS*

Six DEGs and six negative control genes were selected for the qRT-PCR analysis. Among all the non-DEGs, five were expressed in the terminal bud at the flowering stage (*EIL3*, *EIN2L*, *XTH23*, *LNK3*, and *IMP4*), and both the qRT-PCR and RNA-seq data suggested that the RNA-seq and qRT-PCR data were well correlated (Fig. 8). Both *EIN3* and *EIN2L* were expressed in the terminal bud, but their expression did not change significantly. In contrast, *EIL4* was not expressed in the terminal bud (Table S3 and Table S6) and was considered a non-constitutively expressed gene. Among the six DEGs (*SS*, *ABCG26*, *PKSA*, *AGL42*, *FAR2*, and *AGAMOUS*), RNA-Seq and qRT-PCR confirmed significant expression differences between wild-type and Z4 plants. Taken together, the expression patterns from the qRT-PCR analysis were consistent with those obtained from the RNA sequencing.

## Discussion

Cell signaling is a complex process involving many gene networks that is characterized by the non-discrete and non-linear flow of information. Many genes belong to larger gene families and, like soybeans, crops displaying paleopolyploidy have several copies of each gene. Genome complexity leads to high gene redundancy in soybean, which limits the output of mutant screening by forward genetic approaches [19]. A large-scale CRISPR-Cas9 mutagenesis population would be of great value for crop research, and recent studies have generated mutant populations in soybean via pooled transformation of CRISPR-Cas9 libraries [20–22]. To the best of our knowledge, we are the first to edit key-node genes in the ethylene signaling pathway using a multi-sgRNA-in-one strategy. We successfully generated a series of gene edited lines with altered combinations of 15 edited gene targets. We found that two sgRNAs failed to induce mutations in all the T0 plants, six sgRNAs induced low-frequency mutation (< 10%), and one sgRNA induced mutation with a frequency of 67.5%. Although computational models based on DNA sequence have been used to predict the efficiency of sgRNAs, the actual performance of sgRNAs within cells varies greatly [23, 24]. The specific gene editing mutation are often greatly influenced by the efficiency of Cas9, the status of host DNA repair machinery, the DNA damage type, and the structure of DNA repair donor molecules [25]. In the ethylene-insensitive mutant lines of T2 generation, we identified Cas9-free plants carrying targeted mutations, in which the T-DNA was lost while the *EIN2L*, *EIL3*, and *EIL4* genes were all edited. This suggests that the sgRNAs with high frequencies were favored in the transformed plants, and their simultaneous editing may be conducive to the growth and development of soybean.

Ethylene is a stress hormone that temporally accelerates organ abscission in many plant species [26, 27]. Several key components of ethylene signaling, such as *ETR1* and *EIN2*, have been found to induce floral organ shedding in Arabidopsis, but the ethylene-insensitive (*ETR1-1*, *EIN2-1*) mutant lines significantly delayed shedding in Arabidopsis [26–28]. In soybean, treatment with the ethylene inhibitor aminoethoxyvinylglycine increases soybean yield by reducing flower pod shedding [15]. These results suggest that modulating ethylene biosynthesis and signal transduction pathways may be an effective method for creating high-yield germplasm. Following gene editing, we found that *EIL3*, *EIL4*, and *EIN2L* triple mutants expressed earlier flowering (by approximately 7 d) and high-yield (a 65% increase) phenotypes when compared to the wild type plants. Unexpectedly, *EIL4* was not expressed in the terminal bud. In the soybean transcriptome database (https://ngdc.cncb.ac.cn/soyomics/transcriptome/) [29], it was found that *EIL4* (*Glyma.18G018400*) was mainly expressed in seeds during the mature stage R8, suggesting its potential roles in regulation of seed maturation, which was consistent with our result that *EIL4* was not expressed in the terminal bud. Similarly, we also found spatiotemporal expression features of *EIL3* (*Glyma.15G031800*) and *EIN2L* (*Glyma.10G058300*) in the aforementioned database. *EIL3* is specifically highly expressed in floral organs during flowering stage, and this gene is also expressed in pods. *EIN2L* is mainly expressed in floral organs before flowering, as well as in pods and young seeds. Based on this, it is inferred that *EIL3* and *EIN2L* are involved in the regulation of floral organ, pod, and seed development in soybean. Therefore, the simultaneous editing of these three genes may achieve high yield through regulation of floral organ development, pod development, seed development, and seed maturation. *EIL3* and *EIN2L* are expressed in floral organs at different stages, indicating that the editing of them genes may be related to the early flowering of mutant plants.

Comparative transcriptome analysis of the triple mutant and wild type plants revealed 873 DEGs. The number of DEGs was highest in the starch and sucrose metabolism pathway, which contained 33 DEGs. Sucrose synthase (SS), which produces sucrose and uridine diphosphate (UDP), is closely related to flowering and flower development; photosynthetic products mainly transported in the form of sucrose. In pepper (*Capsicum* spp.), sucrose transport into the flowers enlarges the flower sink by suppressing abscission and activating metabolism, resulting in a higher rate of flower set [30]. Furthermore, in cotton (*Gossypium* spp.), GhSusA1 suppression in transgenic plants decreases boll size and seed weight [31]. Based on our results, the expression of 11 DEGs encoding SS in soybean was upregulated by 1.30–4.52 fold compared to nine genes encoding SS that were downregulated by 1.83–3.38-fold (Table 2; Table S4). Overall, compared with the downregulated genes, the amount, expression abundance, and FC of the upregulated genes were dominant. Thus, we speculate that sucrose synthesis and the supply of photoassimilates to axillary buds are enhanced in the triple-mutant plants, which is conducive to flower organ development and increased yield.

**Table 2:** Genes of interest that may be involved in the regulation of flower / seed development

Gene ID	FDR	Log_2_FC	Annotation
*Glyma.04G180300*	2.92E-258	6.12	*At2g26730*
*Glycine_max_newGene_3783*	3.06E-227	5.89	*L9*
*Glyma.03G054100*	2.94E-113	4.52	*SS*
*Glyma.03G053500*	1.41E-80	3.99	*SS*
*Glyma.02G302200*	4.52E-48	2.73	*ABCG26*
*Glyma.11G097900*	5.62E-40	2.63	*PKSB*
*Glyma.07G043300*	1.89E-31	2.43	*CYP704B1*
*Glyma.01G073600*	1.12E-74	2.41	*PKSA*
*Glyma.14G012000*	1.56E-26	2.16	*ABCG26*
*Glyma.08G332100*	1.48E-20	2.16	*ABCG26*
*Glyma.15G018500*	9.43E-37	2.09	*TKPR*
*Glyma.09G104800*	3.06E-19	2.08	*FAR2*
*Glyma.06G184400*	3.94E-19	2.08	*BRI*
*Glyma.19G161100*	6.50E-120	1.80	*AUX28*
*Glyma.20G210400*	3.57E-45	1.33	*IAA14*
*Glyma.10G240500*	2.29E-07	1.24	*AGL42*
*Glyma.11G252300*	1.59E-06	1.14	*MTF1*
*Glyma.15G058000*	4.71E-15	1.06	*JOINTLESS*
*Glyma.13G223300*	5.56E-09	1.04	*AGAMOUS*
*Glyma.07G081300*	1.71E-06	1.02	*MADS3*

Except for gene *Glycine_max_newGene_3783*, the character "*glyma.Wm82.gnm2.*" are omitted before other gene IDs. FDR, false discovery rate; FC, fold change: FPKM of triple mutants divided by FPKM of control; *At2g26730, inactive receptor kinase At2g26730*; *L9, 50S ribosomal protein L9*; *SS, Sucrose synthase*; *ABCG26, ABC transporter G family member 26*; *PKSB, Type III polyketide synthase B*; *CYP704B1, Cytochrome P450 704B1*; *PKSA, Type III polyketide synthase A*; *TKPR, Tetraketide alpha-pyrone reductase*; *FAR2, Fatty acyl-CoA reductase 2*; *BRI, Brassinosteroid Insensitive 1*; *AUX28, Auxin-induced protein AUX28*; *IAA14, Auxin-responsive protein IAA14*; *AGL42, MADS-box protein AGL42*; *MTF1, MADS-box transcription factor 1; JOINTLESS, MADS-box protein JOINTLESS*; *AGAMOUS, Floral homeotic protein AGAMOUS*; *MADS3, Agamous-like MADS-box protein MADS3.* The complete annotations for each DEG are listed in Table S4

The MADS-box genes encode important transcription factors important for flower and fruit development in plants. Significantly, four soybean MADS-box genes were > onefold up-regulated in the triple mutant and their orthologs in other plants have critical roles in flowering and fruit development. *Glyma.10G240500* encodes *AGL42* (Table 2; Table S4) and inactivation of its Arabidopsis ortholog (*AGL42*) produces a non-flowering phenotype [32]. *Glyma.15G058000* encodes *JOINTLESS* (Table 2; Table S4) and inactivation of its *Solanum* spp. ortholog prevents abscission zone formation [33]. *Glyma.13G223300* encodes *AGAMOUS* (Table 2; Table S4) and inactivation of its Arabidopsis ortholog (*AGAMOUS*) decreases the number of mature ovules and increases primordial outgrowth [34]. *Glyma.07G081300* encodes *MADS3* (Table 2; Table S4) and its grapevine (*Vitis vinifera* L.) ortholog is specifically expressed in both male and female flowers and in developing seeds [35]. This suggests that MADS-box genes may affect the yield and flowering period in the triple mutants by regulating flower development.

In maize (*Zea mays*), kernel 44 (*DEK44*) encodes a putative 50S ribosomal protein L9; *DEK44* mutants produce small kernels with embryo-lethal phenotypes [36]. We found that a DEG (*Glycine max newGene_3783*) encoding 50S ribosomal protein L9 was highly upregulated by 5.89-fold in the triple-mutant soybean plants (Table 2; Table S4). This suggests that 50S ribosomal protein L9 may affect the yield in the triple mutants by regulating abscission zones and kernels.

Comparative proteomic analysis of NJCMS2A (soybean cytoplasmic nuclear male sterile line) and NJCMS2B (corresponding maintainer line) at the two-cell pollen stage showed that four proteins were missing in the anthers of NJCMS2A that were present in NJCMS2B, including MADS-box proteins and auxin-inducible protein *AUX28*. Therefore, *AUX28* may play an important role in the regulation of soybean fertility [37]. In cucumber (*Cucumis* spp.), the auxin-responsive gene *IAA14* is involved in sugar-induced parthenocarpic fruit formation [38]. In our study, five DEGs encoding auxin-responsive IAA were found, all of which were upregulated. Among these, *Glyma.19G161100* encodes auxin-induced *AUX28*, with the highest expression abundance, and was upregulated by 1.80-fold. *IAA14* (*Glyma.20G210400*) was also upregulated by 1.33-fold (Table 2; Table S4). Thus, we speculate that the upregulated expression of *AUX28* and *IAA14* enhances soybean fertility.

Flowering is a major event in plant development and is regulated by a series of complex signal transduction pathways. Studies have shown that the *Brassinosteroid Insensitive 1* (*BRI*) mutant *bri1-5* displays delayed flowering [39]. *BRI* is dependent on BR signaling and can promote flowering by inhibiting the expression of *FLOWERING LOCUS C* (*FLC*) [40]. We identified a total of seven DEGs encoding *BRI* in soybean, of which five (*Glyma.06G184400*, *Glyma.18G254000*, *Glyma.18G254500*, *Glyma.18G254600*, and *Glyma.19G081200*) were upregulated by 1.22–2.08 fold, and two (*Glyma.11G083900* and *Glyma.13G177400*) were downregulated by 1.24–1.25 fold compared to wild-type plants; we found no DEGs encoding *FLC* (Table 2; Table S4). In Arabidopsis, plants with the *RLK6* (inactive receptor kinase At2g26730) overexpression mutation bloom earlier than their wild type counterparts [41]. In our study, two DEGs (*glyma.04g180300* and *glyma.06g184400*) encoding At2g26730 orthologs were upregulated by 6.12-fold and 2.08-fold, respectively (Table 2; Table S4). Thus, we speculate that the upregulated expression of *BRI* and *At2g26730* in the triple-mutant soybean activated the BR signal transduction pathway, resulting in earlier flowering when compared to wild type plants.

The exine is the outer wall of pollen and acts as a protective barrier for the male gametophyte. It also mediates pollen-stigma adhesion. In Arabidopsis, *POLYKETIDE SYNTHASE A* (*PKSA*) and *POLYKETIDE SYNTHASE B* (*PKSB*) are specifically and transiently expressed in the tapetal cells of anthers during microspore development. Mutants compromised in *PKS* gene expression display pollen exine layer defects, and a double *PKSA PKSB* male mutant was found to be completely sterile, with no apparent exine [42]. In rapeseed (*Brassica napus*), *BnPKSA*, *BnPKSB*, and *BnACOS5* can form a heterodimer multienzyme complex in tapetal cells that is important for male reproductive development [43]. In *Arabidopsis*, cytochrome P450 (*CYP704B1*) [44], *ABCG26* [45], *TETRAKETIDE alpha-PYRONE REDUCTASE* (*TKPR*) [45], *FAR2* (*FATTY ACID REDUCTASE 2*) [46], *ABCG26* [47, 48] are essential for exine development, and the mutations in these genes cause several phenotypes spanning loss of or an aberrant the exine [44, 46] and/or severely reduced fertility [46–48]. In our studies, *PKSA*, *PKSB*, *CYP704B1*, three *ABCG26*, one *TKPR* (*Glyma.15G018500*), and *FAR2* genes of soybean were over two-fold upregulated in the triple mutant relative to wild-type plants; in addition, a second soybean *TKPR* gene (*Glyma.07G157200*) was upregulated 1.4-fold (Table 2). These data strongly suggest that these soybean genes may help to promote biosynthesis of the pollen exine layer and overall fertility.

## Conclusions

For ethylene signaling, the short-lived *EIN2* and *EIN3* proteins play central roles. To investigate whether differential responses are caused by differential expression of members of the *EIN2* and *EIN3* family transcription factors, all the family members of soybean were identified. By editing key-node genes in the ethylene signaling pathway using CRISPR/Cas9, we obtained a series of gene edited lines with variable combinations of 15 edited target genes. *EIL3*, *EIL4,* and *EIN2L* mutations were prevalent in the T0 gene edited soybean lines. Here, we characterized a T3 soybean line harboring mutations in *EIL3*, *EIL4*, and *EIN2L* (a triple mutant). The T3 generation of the triple mutants had an early flowering time and high-yield phenotype. The yield of the triple mutants was 1.65-fold higher than that of the wild-type controls. We also compared the transcriptomes of the triple-mutant and wild-type terminal buds and identified 873 DEGs. Based on a functional analysis of the main DEGs reported in the literature, a set of DEGs likely involved in the regulation of the early flowering and high-yield phenotype of the triple-mutant line was identified, including *SS*, *AUX28*, *IAA14*, *GIBBERELLIN 20 OXIDASE*, *BRI*, *At2g26730*, *AGL42*, *JOINTLESS*, *AGAMOUS*, *MADS3*, *DEK44*, *PKSA*, *PKSB*, *CYP704B1*, *ABCG26*, *TKPR*, and *FAR2*. These DEGs are mainly involved in the regulation of sugar synthesis, pollen fertility, seed development, and flowering stage; these genes were upregulated in the soybean triple mutants. Overall, our results identify several genes that can be modified to develop earlier-flowering and higher-yielding soybean cultivars.

## Materials and methods

###  Phylogenetic tree analysis of the EIN2/EILs family

All soybean (cultivar Williams 82, W82) *EIN2* and *EIL* (EIN3-like) protein sequences were downloaded from Phytozome13 (https://phytozome-next.jgi.doe.gov/) or the Plant Transcription Factor Database (http://planttfdb.gao-lab.org/) and aligned with those of *A. thaliana*, *Cicer arietinum*, *Glycine soja*, *Lotus japonicus*, *Medicago truncatula*, *Oryza sativa*, *Phaseolus vulgaris*, *Prunus persica*, *Salix purpurea*, and *Vitis vinifera* using the Muscle program within the MEGA 7.0 software package. A phylogenetic tree was constructed using the neighbor-joining (NJ) method with key parameters set as follows: P-distance model, pairwise deletion, and bootstrap = 1,000.

###  Plasmid construction

We designed the sgRNAs of *EIN2* and *EIL* using the CRISPR direct online tool (http://crispr.dbcls.jp/), and their target sequences are listed in Table S7. Pairs of DNA oligonucleotides of the three sgRNAs were synthesized by the Shanghai Sangon Biological Engineering Technology and Service Company (Shanghai, China) and annealed to generate dimers, which were then integrated into Atu3b, Atu3d, Atu6-1, and Atu6-29 sgRNA expression cassettes by overlapping PCR. The target expression cassettes were successfully cloned into pYLCRISPR-Cas9Pubi-B using the Golden Gate cloning method [18]. Cas9 was expressed under the ubiquitin promoter, and positive plasmids were introduced into *Agrobacterium tumefaciens* strain EHA105 for stable transformation (Figure S2).

###  Stable soybean transformation

The soybean cultivar W82 was transformed using the *Agrobacterium*-mediated soybean cotyledon node [24]. T0 generation gene edited soybean plants were obtained following 2–3 months of regeneration and selected with 3–5 mg/L glufosinate-supplemented medium. Gene edited plants were confirmed (gene edited T0 and T1 plants) by spraying the total plants with 900 mg/L of glufosinate ammonium. Gene edited glufosinate-resistant (GR) soybean materials were then subject to amplicon deep sequencing of all genome-edited sites.

### Amplicon deep sequencing of all genome-edited sites

The amplicon PCR products of T0 lines for next-generation sequencing were obtained through two steps. First, we performed 25-μL reactions each with soybean genomic DNA to amplify the sgRNA in the target regions. Second, Illumina adaptors (i7 and i5 primers) were attached and to barcode samples in a 25-μL reactions volume using 2 μL of the product from the first PCR step. The sequences obtained by multiplex amplicon sequencing in the single nucleotide polymorphism (SNP) genotyping are listed in Table S7. The synthesis of primers and amplicon deep SNP sequencing of all genome-edited sites were performed by Shanghai Sangon Biological Engineering Technology and Service Company (Shanghai, China). The raw sequencing data were filtered for quality control purposes with default parameters [49]. All merged reads were subsequently aligned to the reference using the BWA-MEM algorithm [50].

### Phenotypic characterization of wild-type and the Z4 triple mutant plants

In the constitutive triple-response mutant lines for ethylene, we identified Cas9-free lines carrying targeted mutations, in which the T-DNA was lost while the *EIN2L*, *EIL3*, and *EIL4* genes were transmitted. A pot experiment was conducted outdoors at Jilin Normal University, Siping City, Jilin Province, between 2019 and 2021 using the T2 generation seeds of the W82 and its *EIN2L, EIL3,* and *EIL4* homozygous triple mutants (abbreviated as experiment number Z4). Plastic pots with an upper diameter, lower diameter, and height of 24, 17, and 20 cm, respectively, were used for cultivation. Twenty 1–5-cm-diameter holes were made at the bottom of the pots, and each pot was filled with approximately 6.0 kg of the cultivation medium (a 3:1 mixture of soil and vermiculite). The spacing between pots was uniformly and consistently 30 cm. To reduce border effects, potted W82 were placed around the experimental potted plants. W82 and Z4 plants were randomly arranged on the inner side of the border row. Three W82 or Z4 seeds were sown in each of the 30 pots (sowing depth = 3.0 cm) and one seedling was retained after germination. When the first flower and pod were observed on each plant, the corresponding flowering time and seed-setting stage were recorded for 10 individual plants, respectively. At the seed-maturity stage, physiological indexes such as plant height, above-ground dry weight, pod number, bean number, 100-seed weight, and yield per plant were measured for at least 10 individual plants. All the developmental periods were determined according to the literature [51].

### Transcriptome sequencing of wild type and gene-edited soybean

Z4 and wild-type plants were grown under the conditions described for the phenotypic analyses above. Also, some of them were used to carry out RNA extraction and transcriptome sequencing. When the potted wild-type soybean flowered (approximately 35 d after cultivation), the terminal buds of the wild-type control and the Z4 plants were sampled. To investigate gene expression changes between the wild-type and gene-edited soybean, we constructed six DGE profiling libraries: CK-1, CK-2, CK-3, Z4-1, Z4-2, and Z4-3. Thus, our experiment included two treatments (WT and triple mutation), each treatment included three replicates, and each replicate included five terminal buds. Total RNA was extracted from the flower bud samples using the RNA EasySpin Isolation System (Aidlab Biotech, Beijing, China), and RNA pretreatment and mRNA sequencing were carried out as previously described [52]. Sequencing was performed using a HiSeq 4000 system (Illumina, San Diego, CA, USA), and raw transcriptome data were deposited in the Sequence Read Archive (https://www.ncbi.nlm.nih.gov/SRA) under BioProject number PRJNA774476.

### Bioinformatics analysis of transcriptome data

High-quality clean data were obtained by filtering the raw data, which removed low-quality adapter sequences and reads. Cleaned data were then aligned to the soybean reference genome (https://soybase.org/data/public/Glycine_max/Wm82.gnm2.DTC4/) to generate the mapped data. ‘HISAT2’ (http://ccb.jhu.edu/software/hisat2/index.shtml) was used to map the RNA-seq reads [53], and ‘StringTie’ (https://ccb.jhu.edu/software/stringtie/index.shtml) was used to assemble the mapped reads [54]. Gene function was annotated based on Nr (NCBI non-redundant protein sequences), Nt (NCBI non-redundant nucleotide sequences), Pfam (Protein family), KOG/COG (Clusters of Orthologous Groups of proteins), Swiss-Prot (a manually annotated and reviewed protein sequence database), KO (KEGG Ortholog), and GO (Gene Ontology) databases. FPKM (fragments per kilobase of transcript per million fragments mapped) normalization was applied to measure the expression level of transcripts by StringTie using the maximum flow algorithm. DEGs were identified using ‘DESeq2’ [55]. The criteria for DEGs were set as FC ≥ 2 and FDR < 0.01. FC refers to the ratio of gene expression in the two samples; and FDR refers to the adjusted *p*-value, which was used to measure the significance of differences. Gene set enrichment analysis [56] was performed on all genes based on their expression levels, and the gene sets of KEGG pathways and GO terms for BP, CC, and MF were targeted as the gene sets of interest. Genes from each group were used as the background gene set, and enriched gene sets were identified where *p* < 0.001 and FDR < 0.05.

### qRT-PCR

Using the same samples that were used for RNA-seq analysis, the RNA Easy Spin Isolation System (Aidlab Biotech, Beijing, China) was utilized to extract total RNA from the soybean plants according to the manufacturer’s instructions. In total, 12 DEGs and non-DEGs were chosen for qRT-PCR analysis, and primer designing and qRT-PCR analysis were carried out as previously described [57]. The detailed primer sequences are listed in Table S8. Beta actin (*ACTB*) was used as a reference gene in the qRT-PCR assay, and the 2^−ΔΔCt^ method was used to calculate the FCs of the chosen genes [58].

### Statistical analysis

Analysis of variance (ANOVA) or generalized linear modeling (GLM) in SAS version 8.01 (SAS Institute, Inc., Cary, NC, USA) were used to test for differences in phenotypic data between multiple groups. The means of the data obtained from the control and gene-edited plants were compared using the least significant difference test at 5% significance level.

### Supplementary Information

Below is the link to the electronic supplementary material.

**Additional file 1: Figure S1.** Differentially expressed transcripts in KEGG pathway of plant hormone signal transduction.

**Additional file 2: Figure S2.** Schematic diagram of the T-DNA region of the targeting vector.

**Additional file 3: Table S1.** Sequencing data statistics.

**Additional file 4: Table S2.** Statistics on data mapping.

**Additional file 5: Table S3.** Differentially expressed genes.

**Additional file 6: Table S4.** Annotation of differentially expressed genes.

**Additional file 7: Table S5.** KEGG enrichment analysis of differentially expressed genes.

**Additional file 8: Table S6.** FPKM value of all expressed genes.

**Additional file 9: Table S7.** Target sequence and SNP genotyping by multiplex amplicon sequencing.

**Additional file 10: Table S8.** Primers sequence information used in qRT-PCR.

## Data Availability

The original contributions presented in the study are publicly available. All raw transcriptome data were deposited in the sequence read archive (accession no. PRJNA774476).

## References

[1] Guzman P, Ecker JR (1990). Exploiting the triple response of Arabidopsis to identify ethylene-related mutants. Plant Cell.

[2] Qiao H, Shen Z, Huang SSC, Schmitz RJ, Urich MA, Briggs SP, Ecker JR (2012). Processing and subcellular trafficking of ER-tethered EIN2 control response to ethylene gas. Science.

[3] Leonetti P, Zonno MC, Molinari S, Altomare C (2017). Induction of SA-signaling pathway and ethylene biosynthesis in Trichoderma harzianum-treated tomato plants after infection of the root-knot nematode Meloidogyne incognita. Plant Cell Rep.

[4] Lei G, Shen M, Zhi-Gang LI, Zhang BO, Duan KX, Wang N, Cao YR, Zhang WK, Biao MA, Ling HQ (2011). Ein2 regulates salt stress response and interacts with a MA3 domain-containing protein ECIP1 in Arabidopsis. Plant Cell Environ.

[5] Binder BM (2004). Short-Term growth responses to ethylene in Arabidopsis seedlings are EIN3/EIL1 independent. Plant Physiol.

[6] Ann A, Ying Z, Lin Y, Liu J, Zhang Z, Tang Z (2017). Ethylene improves root system development under cadmium stress by modulating superoxide anion concentration in Arabidopsis thaliana. Front Plant Sci.

[7] Wang L, Zhang Z, Zhang F, Shao Z, Zhao B, Huang A, Tran J, Hernandez F, Qiao H (2021). EIN2-directed histone acetylation requires EIN3-mediated positive feedback regulation in response to ethylene. Plant Cell.

[8] Shibuya K, Barry KG, Ciardi JA, Loucas HM, Underwood BA, Nourizadeh S, Ecker JR, Klee HJ, Clark DG (2004). The central role of phein2 in ethylene responses throughout plant development in Petunia. Plant Physiol.

[9] Liu C, Ma T, Yuan D, Zhou Y, Long Y, Li Z, Dong Z, Duan M, Yu D, Jing Y, Bai X, Wang Y, Hou Q, Liu S, Zhang JS, Chen SY, Li D, Liu X, Li Z, Wang W, Li J, Wei X, Ma B, Wan X (2022). The OsEIL1-OsERF115-target gene regulatory module controls grain size and weight in rice. Plant Biotechnol J.

[10] Zhao H, Duan KX, Ma B, Yin CC, Hu Y, Tao JJ, Huang YH, Cao WQ, Chen H, Yang C, Zhang ZG, He SJ, Zhang WK, Wan XY, Lu TG, Chen SY, Zhang JS (2020). Histidine kinase MHZ1/OsHK1 interacts with ethylene receptors to regulate root growth in rice. Nat Commun.

[11] Chao Y, Ma B, He SJ, Xiong Q, Zhang JS (2015). Mhz6/OsEIL1 and OsEIL2 regulate ethylene response of roots and coleoptiles and negatively affect salt tolerance in rice. Plant Physiol.

[12] Hiraga S, Sasaki K, Hibi T, Yoshida H, Uchida E, Kosugi S, Kato T, Mie T, Ito H, Katou S, Seo S, Matsui H, Ohashi Y, Mitsuhara I (2009). Involvement of two rice ETHYLENE INSENSITIVE3-LIKE genes in wound signaling. Mol Genet Genomics.

[13] Cheng Y, Liu J, Yang X, Ma R, Liu C (2013). Construction of ethylene regulatory network based on the phytohormones related gene transcriptome profiling and prediction of transcription factor activities in soybean. Acta Physiol Plant.

[14] Cheng Y, Liu J, Yang X, Ma R, Liu C, Liu Q (2013). RNA-seq analysis reveals ethylene-mediated reproductive organ development and abscission in soybean (Glycine max L. Merr.). Plant Mol Biol Rep.

[15] Cheng Y, Zhang Q, Liu J, Zhang H, Zhang C (2014). Studies on pollen fertility regulated by exogenous ethylene in soybean (Glycine max L. Merr). J Zhejiang Univ (Agric. & Life Sci.).

[16] Solano C, Sesma B, Alvarez M, Humphrey TJ, Gamazo C (1998). Discrimination of strains of salmonella enteritidis with differing levels of virulence by an in vitro glass adherence test. J Clin Microbiol.

[17] Chao Q, Rothenberg M, Solano R, Roman G, Terzaghi W, Ecker JR (1997). Activation of the ethylene gas response pathway in Arabidopsis by the nuclear protein ETHYLENE-INSENSITIVE3 and related proteins. Cell.

[18] Ma X, Liu Y (2016). CRISPR/Cas9-based multiplex genome editing in monocot and dicot plants. Curr Protoc Mol Biol.

[19] Shoemaker RC, Schlueter J, Doyle JJ, Shoemaker RC, Schlueter J, Doyle JJ (2006). Paleopolyploidy and genome duplication in soybean and other legumes. Curr Opin Plant Biol.

[20] Zhang F, Wang L, Qi B, Zhao B, Ko EE, Riggan ND, Chin K, Qiao H (2017). EIN2 mediates direct regulation of histone acetylation in the ethylene response. Proc Natl Acad Sci USA.

[21] Bai M, Yuan JH, Kuang HQ, Gong PP, Li SN, Zhang ZH, Liu B, Sun JF, Yang MX, Yang L, Wang D, Song S, Guan YF (2020). Generation of a multiplex mutagenesis population via pooled CRISPR-Cas9 in soybean. Plant Biotechnol J.

[22] Bai M, Yuan C, Kuang H, Sun Q, Hu X, Cui L, Lin W, Peng C, Yue P, Song S, Guo Z, Guan Y (2022). Combination of two multiplex genome-edited soybean varieties enables customization of protein functional properties. Mol Plant.

[23] Ma S, Wang A, Chen X, Zhang T, Xing W, Xia Q (2021). Deep sequencing reveals the comprehensive CRISPR-Cas9 editing spectrum in Bombyx mori. CRISPR J.

[24] Xiao P, Liu Y, Cao Y (2019). Overexpression of *G10-EPSPS* in soybean provides high glyphosate tolerance. J Integr Agr.

[25] Liang X, Potter J, Kumar S, Ravinder N, Chesnut JD (2017). Enhanced CRISPR/Cas9-mediated precise genome editing by improved design and delivery of gRNA, Cas9 nuclease, and donor DNA. J Biotechnol.

[26] Niederhuth CE, Cho SK, Seitz K, Walker JC (2013). Letting go is never easy: abscission and receptor-like protein kinases. J Integr Plant Biol.

[27] Butenko MA, Patterson SE, Grini PE, Stenvik GE, Aalen RB (2003). Inflorescence deficient in abscission controls floral organ abscission in Arabidopsis and identifies a novel family of putative ligands in plants. Plant Cell.

[28] Chang C, Kwok SF, Bleecker AB, Meyerowitz EM (1993). Arabidopsis ethylene response gene *ETR1*: similarity of product to two-component regulators. Science.

[29] Liu Y, Du H, Li P, Shen Y, Peng H, Liu S, Zhou GA, Zhang H, Liu Z, Shi M, Huang X, Li Y, Zhang M, Wang Z, Zhu B, Han B, Liang C, Tian Z (2020). Pan-genome of wild and cultivated soybeans. Cell.

[30] Aloni B, Karni L, Zaidman Z, Schaffer AA (1997). The relationship between sucrose supply, sucrose-cleaving enzymes and flower abortion in pepper. Ann Bot.

[31] Jiang Y, Guo W, Zhu H, Ruan YL, Zhang T (2012). Overexpression of GhSusa1 increases plant biomass and improves cotton fiber yield and quality. Plant Biotechnol J.

[32] Dorca-Fornell C, Gregis V, Grandi V, Coupland G, Kater MM (2011). The Arabidopsis SOC1-like genes AGL42, AGL71 and AGL72 promote flowering in the shoot apical and axillary meristems. Plant J.

[33] Mao L, Begum D, Chuang HW, Budiman MA, Szymkowiak EJ, Irish EE, Wing RA (2000). JOINTLESS is a MADS-box gene controlling tomato flower abscission zone development. Nature.

[34] Western TL, Haughn GW (2010). BELL1 and AGAMOUS genes promote ovule identity in Arabidopsis thaliana. Plant J.

[35] Boss PK, Sensi E, Hua C, Davies C, Thomas MR (2002). Cloning and characterization of grapevine (Vitis vinifera L) MADS-box genes expressed during inflorescence and berry development. Plant Sci..

[36] Qi W, Lu L, Huang S, Song R (2019). Maize Dek44 encodes mitochondrial ribosomal protein L9 and is required for seed development. Plant Physiol.

[37] Zeng W, Yang S, Yu D, Gai J (2007). A comparative study on anther proteomics between cytoplasmic-nuclear male-sterile line NJCMS2A and its maintainer of soybean. Acta Agron Sin.

[38] Wang M, Su L, Cong Y, Chen J, Qi X (2021). Sugars enhance parthenocarpic fruit formation in cucumber by promoting auxin and cytokinin signaling. Sci Hortic.

[39] Li J, Li Y, Chen S, An L (2010). Involvement of brassinosteroid signals in the floral-induction network of Arabidopsis. J Exp Bot.

[40] Domagalska MA, Schomburg FM, Amasino RM, Vierstra RD, Nagy F, Davis SJ (2007). Attenuation of brassinosteroid signaling enhances FLC expression and delays flowering. Development.

[41] Yang M, Han Y, Habaike A, Wang C (2017). Study of RLK6, one member of leucine-rich repeat receptor-like kinases (LRR-RLKs) subfamily genes, on process of flowering in Arabidopsis. J Nucl Agr Sci.

[42] Kim SS, Grienenberger E, Lallemand B, Colpitts CC, Kim SY, Souza CA, Geoffroy P, Heintz D, Krahn D, Krahn D, Kaiser M, Kombrink E, Heitz T, Suh DY, Legrand M, Douglas CJ (2010). LAP6/POLYKETIDE SYNTHASE A and LAP5/POLYKETIDE SYNTHASE B encode hydroxyalkyl α-pyrone synthases required for pollen development and sporopollenin biosynthesis in Arabidopsis thaliana. Plant Cell.

[43] Qin M, Tian T, Xia S, Wang Z, Song L, Yi B, Wen J, Shen J, Ma C, Fu T, Tu J (2016). Heterodimer formation of BnPKSA or BnPKSB with BnACOS5 constitutes a multienzyme complex in tapetal cells and is involved in male reproductive development in Brassica napus. Plant Cell Physiol.

[44] Dobritsa AA, Shrestha J, Morant M, Pinot F, Matsuno M, Swanson R, Moller BL, Preuss D (2009). CYP704B1 is a long-chain fatty acid ω-hydroxylase essential for sporopollenin synthesis in pollen of Arabidopsis. Plant Physiol.

[45] Dobritsa AA, Geanconteri A, Shrestha J, Carlson A, Kooyers N, Coerper D, Urbanczyk-Wochniak E, Bench BJ, Sumner LW, Swanson R, Preuss D (2011). A large-scale genetic screen in Arabidopsis to identify genes involved in pollen exine production. Plant Physiol.

[46] Thuy TPD, Anders SC, Sten S, Per H (2016). Biochemical characteristics of AtFAR2, a fatty acid reductase from Arabidopsis thaliana that reduces fatty acyl-CoA and -ACP substrates into fatty alcohols. Acta Biochim Pol.

[47] Zhao G, Shi J, Liang W, Xue F, Luo Q, Zhu L, Qu G, Chen M, Schreiber L, Zhang D (2015). Two ATP binding cassette g transporters, rice ATP binding cassette G26 and ATP binding cassette G15, collaboratively regulate rice male reproduction. Plant Physiol.

[48] Quilichini TD, Friedmann MC, Samuels AL, Douglas CJ (2010). ATP-binding cassette transporter G26 is required for male fertility and pollen exine formation in Arabidopsis. Plant Physiol.

[49] He B, Zhu R, Yang H, Lu Q, Lang J (2020). Assessing the impact of data preprocessing on analyzing next generation sequencing data. Front Bioeng Biotech.

[50] Jung Y, Han D (2022). BWA-MEME: BWA-MEM emulated with a machine learning approach. Bioinformatics.

[51] Fehr WR, Caviness CE (1977). Stages of soybean development. Iowa State Univ Coop Ext Ser Spec Rep.

[52] Cheng Y, Zhang Y, Liu C, Ai P, Liu J (2018). Identification of genes regulating ovary differentiation after pollination in hazel by comparative transcriptome analysis. BMC Plant Biol.

[53] Kim D, Langmead B, Salzberg SL (2015). HISAT: a fast spliced aligner with low memory requirements. Nat Methods.

[54] Pertea M, Pertea GM, Antonescu CM, Chang TC, Mendell JT, Salzberg SL (2015). StringTie enables improved reconstruction of a transcriptome from RNA-seq reads. Nat Biotechnol.

[55] Love MI, Huber W, Anders S (2014). Moderated estimation of fold change and dispersion for RNA-seq data with DESeq2. Genome Biol.

[56] Tan G, Lenhard B (2016). TFBSTools: an R/bioconductor package for transcription factor binding site analysis. Bioinformatics.

[57] Liu JF, Liu JY, Zhang XZ, Wei H, Ren JH, Peng C, Cheng YQ (2021). Pollen tube in hazel grows intermittently: role of Ca^2+^ and expression of auto-inhibited Ca^2+^ pump. Sci Hortic.

[58] Schmittgen TD, Livak KJ (2008). Analyzing real-time PCR data by the comparative CT method. Nat Protoc.

