# Supplementary material for: Multiplex CRISPR-Cas9 knockout of EIL3, EIL4, and EIN2L advances soybean flowering time and pod set
Source: BMC Plant Biol. 2023 Oct 27;23:519. doi: 10.1186/s12870-023-04543-x (PMC10604859; doi:10.1186/s12870-023-04543-x)
Supplement: Supplementary file 1 — Additional file 1: Figure S1. Differentially expressed transcripts in KEGG pathway of plant hormone signal transduction. [file 12870_2023_4543_MOESM1_ESM.docx]

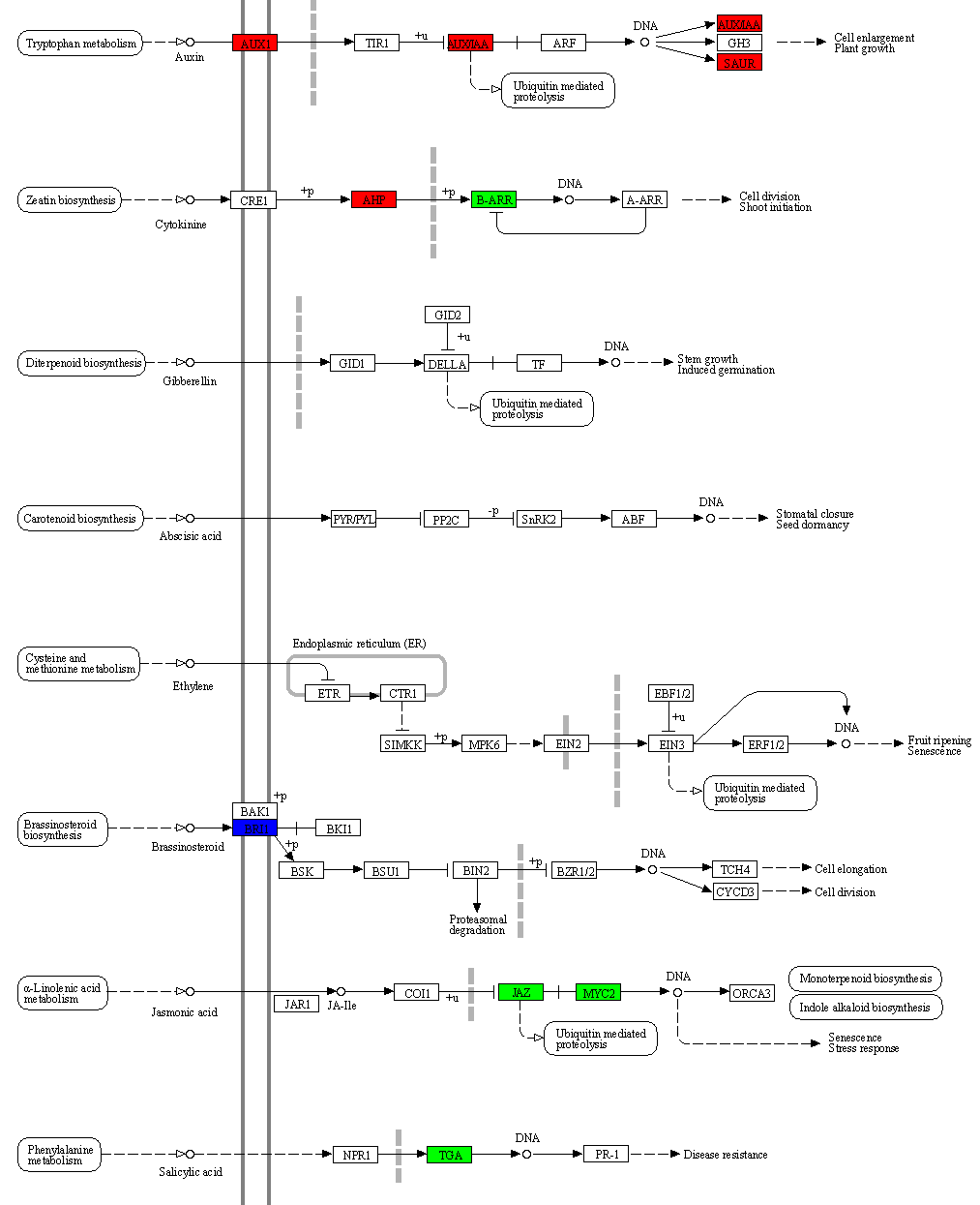


**Figure S1. Differentially expressed transcripts in KEGG pathway of plant hormone signal transduction.**
