# Supplementary material for: Multiplex CRISPR-Cas9 knockout of EIL3, EIL4, and EIN2L advances soybean flowering time and pod set
Source: BMC Plant Biol. 2023 Oct 27;23:519. doi: 10.1186/s12870-023-04543-x (PMC10604859; doi:10.1186/s12870-023-04543-x)
Supplement: Supplementary file 2 — Additional file 2: Figure S2. Schematic diagram of the T-DNA region of the targeting vector. [file 12870_2023_4543_MOESM2_ESM.docx]

**
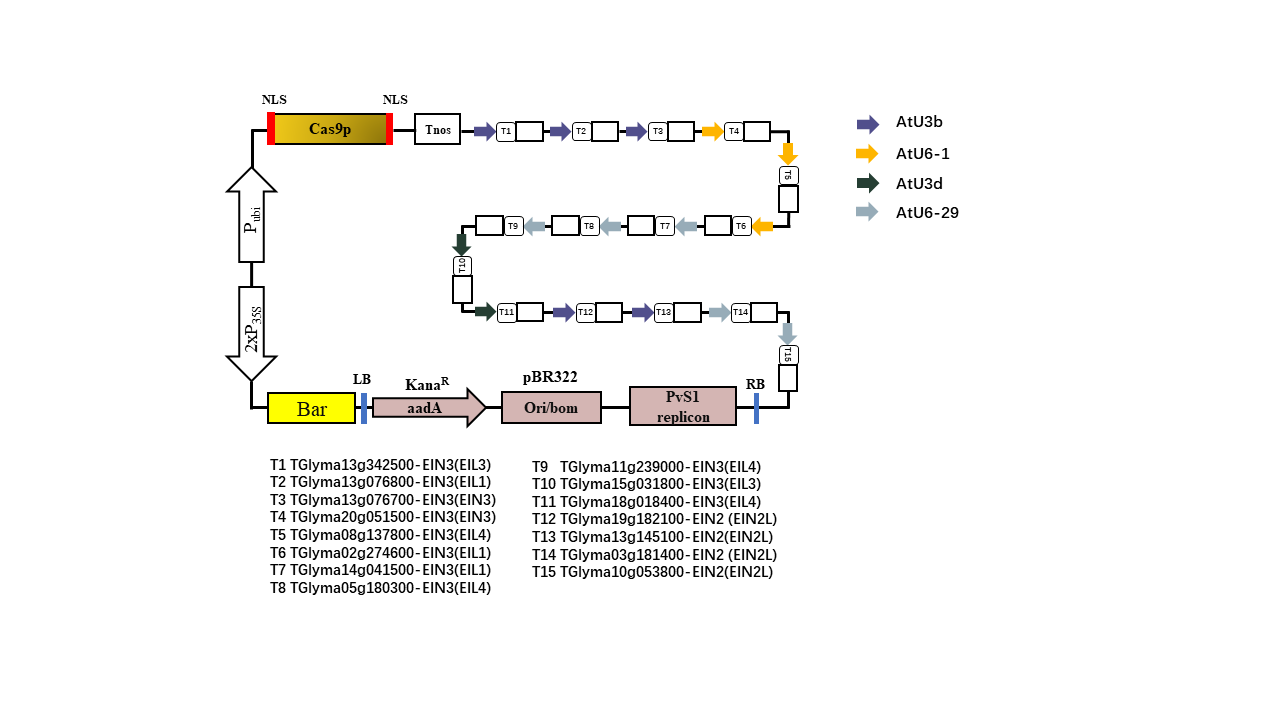
Figure S2** Schematic diagram of the T-DNA region of the targeting vector.

UBI promoter indicates a constitutive strong promoter in maize. U3b, U3d, U6-1, and U6-29 indicates the Arabidopsis U3b, U3d, U6-1, and U6-29 promoter. Sg indicates the single-guide RNA. T1, T2, and T3 indicate the target sequences of *EIL3, EIL4*, and *EIN2L*, respectively. The bar indicates the phosphinothricin-resistance gene. LB and RB indicate the left border and right border, respectively.
