# Supplementary material for: Multiplex CRISPR-Cas9 knockout of EIL3, EIL4, and EIN2L advances soybean flowering time and pod set
Source: BMC Plant Biol. 2023 Oct 27;23:519. doi: 10.1186/s12870-023-04543-x (PMC10604859; doi:10.1186/s12870-023-04543-x)
Supplement: Supplementary file 3 — Additional file 3: Table S1. Sequencing data statistics. [file 12870_2023_4543_MOESM3_ESM.docx]

Table S1 Sequencing data Statistics

| **Samples** | **Clean reads** | **Clean bases** | **GC Content** | **%≥Q30** |
| --- | --- | --- | --- | --- |
| CK1 | 28,260,214 | 8,427,594,880 | 44.90% | 94.04% |
| CK2 | 21,832,349 | 6,497,892,506 | 44.91% | 93.57% |
| CK3 | 27,705,399 | 8,242,809,588 | 44.79% | 94.28% |
| Z4-1 | 29,885,699 | 8,900,158,448 | 44.65% | 93.80% |
| Z4-2 | 28,251,973 | 8,407,031,660 | 44.62% | 94.05% |
| Z4-3 | 33,656,721 | 10,023,100,876 | 44.74% | 94.62% |

Note: (1) Samples: Sample name;

(2) Clean reads: Counts of clean PE reads;

(3) Clean bases: total base number of Clean Data;

(4) GC content: Percentage of G, C in clean data.

(5) ≥Q30%: Percentage of bases with Q-score no less than Q30.
