# Supplementary material for: Multiplex CRISPR-Cas9 knockout of EIL3, EIL4, and EIN2L advances soybean flowering time and pod set
Source: BMC Plant Biol. 2023 Oct 27;23:519. doi: 10.1186/s12870-023-04543-x (PMC10604859; doi:10.1186/s12870-023-04543-x)
Supplement: Supplementary file 4 — Additional file 4: Table S2. Statistics on data mapping. [file 12870_2023_4543_MOESM4_ESM.docx]

Table S2 Statistics on data mapping

| **Sample** | **Total Reads** | **Mapped Reads** | **Uniq Mapped Reads** | **Multiple Map Reads** | **Reads Map to '+'** | **Reads Map to '-'** |
| --- | --- | --- | --- | --- | --- | --- |
| CK1 | 56,520,428 | 54,350,442 (96.16%) | 52,970,299 (93.72%) | 1,380,143 (2.44%) | 27,071,388 (47.90%) | 27,121,676 (47.99%) |
| CK2 | 43,664,698 | 41,781,869 (95.69%) | 40,711,060 (93.24%) | 1,070,809 (2.45%) | 20,807,139 (47.65%) | 20,848,094 (47.75%) |
| CK3 | 55,410,798 | 53,154,328 (95.93%) | 51,767,421 (93.42%) | 1,386,907 (2.50%) | 26,467,847 (47.77%) | 26,511,946 (47.85%) |
| Z4-1 | 59,771,398 | 57,264,014 (95.81%) | 55,842,695 (93.43%) | 1,421,319 (2.38%) | 28,519,755 (47.71%) | 28,571,626 (47.80%) |
| Z4-2 | 56,503,946 | 54,033,574 (95.63%) | 52,693,431 (93.26%) | 1,340,143 (2.37%) | 26,916,264 (47.64%) | 26,962,070 (47.72%) |
| Z4-3 | 67,313,442 | 64,479,445 (95.79%) | 62,869,264 (93.40%) | 1,610,181 (2.39%) | 32,113,910 (47.71%) | 32,174,054 (47.80%) |

Note: Sample: sample ID in system;

Total Reads: Counts of Clean Reads, counted as single end;

Mapped Reads: Counts of mapped reads and the proportion of that in clean data;

Uniq Mapped Reads: Counts of reads mapped to a unique position on reference genome and proportion of that in clean data;

Multiple Mapped Reads: Counts of reads mapped to multiple positions on reference genome and proportion of that in clean data;

Reads Map to '+': Counts of reads mapped to the sense chain and the proportion of that in clean data;

Reads Map to '-': Counts of reads mapped to antisense chain and proportion of that in clean data.
