# Supplementary material for: Multiplex CRISPR-Cas9 knockout of EIL3, EIL4, and EIN2L advances soybean flowering time and pod set
Source: BMC Plant Biol. 2023 Oct 27;23:519. doi: 10.1186/s12870-023-04543-x (PMC10604859; doi:10.1186/s12870-023-04543-x)
Supplement: Supplementary file 10 — Additional file 10: Table S8. Primers sequence information used in qRT-PCR. [file 12870_2023_4543_MOESM10_ESM.docx]

Table S8 Primers sequence information used in qRT-PCR

| **Gene ID** | Annotation | Primer sequences |
| --- | --- | --- |
| ***Glyma.15G031800*** | *EIL3* | F：5'CGAAGAAATTGGAGCTGATG 3'  R: 5'GCTCCAATTCTTCTGCTTCA 3' |
| ***Glyma.18G018400*** | *EIL4* | F: 5'AGGAGAAGCGGCAGAAACAG 3'  R: 5'GGCGTTGCAGACCTCCATGA 3' |
| ***Glyma.10G058300*** | *EIN2L* | F: 5'TGGCCTTCACACTTATCTTT 3'  R: 5'AGCATAATCACCGAAAGTTC 3' |
| glyma.Wm82.gnm2.Glyma.03G054100 | *SS* | F: 5'AGTTTCAGAGGGGACGACAC  R: 5'CCCTTGGAAGTGTCTCATCA3' |
| glyma.Wm82.gnm2.Glyma.02G302200 | *ABCG26* | F: 5'GAAGGAAAGAGAAGATGAGA 3'  R: 5'TCTTGGTTGAAACTTGAATC 3' |
| glyma.Wm82.gnm2.Glyma.01G073600 | *PKSA* | F: 5'CCTTCCCTAGCCAAATCATC 3'  R: 5'TATGGTTGGTGAGCCCTCTG 3' |
| glyma.Wm82.gnm2.Glyma.10G240500 | *AGL42* | F: 5'AGGATACAACAAGCCGTCAA 3'  R: 5'CGGTATCGTTCCAAAATCTT 3' |
| glyma.Wm82.gnm2.Glyma.09G104800 | *FAR2* | F: 5'AATGAACCAAAAGCCAGATA 3'  R: 5'GGAGCTAAAAAACTTCATCA 3' |
| glyma.Wm82.gnm2.Glyma.13G223300 | *AGAMOUS* | F: 5'ACCCATCCATGTCAGTTTCT 3'  R: 5'CGGCTAGAGAAAACAATGAG 3' |
| glyma.Wm82.gnm2.Glyma.13G103100 | *XTH23* | F: 5'TTGAGATAACATGGGGTGAT 3'  R: 5'TCCTAGAACGAAATCCAGAG 3' |
| glyma.Wm82.gnm2.Glyma.11G158200 | *LNK3* | F: 5'GAACCGATGAATGACACTTT 3'  R: 5'CTGCTCAAATATGTCCAAGA3' |
| glyma.Wm82.gnm2.Glyma.11G152600 | *IMP4* | F: 5'TACGAAAAGAAGCGCAAAAT 3'  R: 5'TTCCGCAGCATAAGCATACT 3' |
| Glyma.02g091900 | *Actin* | F: 5'GACCTTCAACACCCCTGCT3'  R: 5'GTGGGAGTGCATAACCCTC3' |

EIL3, Ethylene insensitive 3; EIL4, Ethylene insensitive 4； EIN2L , Ethylene insensitive 2 like; SS, sucrose synthase；ABCG26， ABC transporter G family member 26；PKSA, Type III polyketide synthase A; AGL42, MADS-box protein AGL42; FAR2，FATTY ACID REDUCTASE2；AGAMOUS, Floral homeotic protein AGAMOUS；XTH23, xyloglucan endotransglucosylase/hydrolase protein 23；LNK3， Protein LNK3； IMP4, U3 small nucleolar ribonucleoprotein protein IMP4.
